# Tension-type headache in the Emergency Department Diagnosis and misdiagnosis: The TEDDi study

**DOI:** 10.1038/s41598-020-59171-4

**Published:** 2020-02-12

**Authors:** D. García-Azorín, M. Farid-Zahran, M. Gutiérrez-Sánchez, M. N. González-García, A. L. Guerrero, J. Porta-Etessam

**Affiliations:** 1 Headache Unit, Neurology Department, Hospital Clínico Universitario de Valladolid, Valladolid, Spain; 2 Internal Medicine Department, Hospital Clínico Universitario San Carlos, Madrid, Spain; 3 Headache Unit, Neurology Department, Hospital Clínico Universitario San Carlos, Madrid, Spain; 4 Institute for Biomedical Research of Salamanca (IBSAL), Salamanca, Spain; 5 Department of Medicine, University of Valladolid, Valladolid, Spain

**Keywords:** Diagnosis, Headache, Epidemiology

## Abstract

Headache is a common reason to visit the emergency department (ED). Tension-type headache (TTH) is the commonest headache. The diagnosis of TTH implies a mild condition, with no need for special tests. We evaluated the use of the International Classification of Headache Disorders (ICHD) criteria for TTH in the ED. We performed a cross-sectional study including all ED patients with a definite TTH diagnosis in their discharge report for 2.5 years. We evaluated whether the ICHD criteria for TTH were referenced and met. We analysed discrepancies concerning anamnesis or prior history and reclassified patients. A total of 211 out of 2132 patients fulfilled the criteria (9.9%). Only five patients fulfilled TTH criteria. Criteria A-D were referenced in 60–84% of patients and met in 16–74% of these patients. Anamnesis was discrepant in 87.5% as was prior history in 20.8%. After re-reclassification, 21 patients fulfilled the criteria for TTH (five) or probable TTH (16). In 106 patients, another headache was diagnosed, with migraine in 40 (18.9%), secondary headache in 64 (30.3%), and a life-threatening disorder in 13 (6.1%). In our sample, TTH was overdiagnosed. Only a minority of patients fulfilled the ICHD criteria. Inconsistencies in prior medical history or anamnesis were frequent.

## Introduction

Tension-type headache (TTH) is the most common primary headache disorder^1,2^. The prevalence of TTH is estimated to be between 30 and 70% of the general population according to different studies^2^. The diagnostic criteria proposed by the International Classification of Headache Disorders (ICHD) have remained unaltered since the first edition in 1988^3–6^. The criteria are based on bilateral, oppressive and mild pain, without typical migraine features and with no better explanation^6^. Given the mild nature of the disorder, few patients seek assistance, and in headache unit-based series, it is not a frequent diagnosis, accounting for 16% of all diagnoses^7^. On the other hand, it is not uncommon for migraine patients to be erroneously diagnosed with TTH^8^.

Headache is one of the main reasons for consultation in the emergency department (ED). It seems remarkable that in some ED-based series, TTH diagnosis accounts for up to 25–33% of all headache visits;^9,10^ particularly when other series are performed by neurologists or using ICHD criteria diagnosis, TTH represents only 1–6% of total headache patients^11–15^. In the ED setting, secondary headache detection represents the main priority. TTH is particularly threatening, as its typical phenotype is relatively unspecific, and many secondary headaches may mimic it.

The use and knowledge of the ICHD criteria in the ED setting may be difficult. Most clinicians use red flag lists to rule out secondary headaches^16^. However, we hypothesize that TTH is probably overdiagnosed in the ED setting, which might represent a risk for patients with nondetected secondary headaches. Establishing TTH diagnosis might be dangerous, as it implies that the patient has a harmless disorder.

In this study, the first objective was to analyse the percentage of patients who fulfilled the ICHD^6^ criteria for tension-type headache and the percentage of patients presenting each of the different criteria. The second objective was to analyse the presence of data in the discharge reports that contradicted TTH diagnosis, such as relevant prior medical data, atypical symptoms or abnormal findings in the examination. The third objective was to analyse whether patients could be re-classified as having other headache disorders by using the ICHD-3 criteria.

## Patients and Methods

This is an observational study with a cross-sectional design. Our study population included patients who visited the emergency department due to headache. The study was performed according to the Strengthening the Reporting of Observational Studies in Epidemiology (STROBE) guidelines^17^.

The study took place at the ED of the Clínico San Carlos University Hospital, Madrid (Spain), a third-level hospital with a reference population of 700.000 people. The study period was between January 2012 and July 2014.

### Eligibility

The inclusion criteria were as follows: 1) patients visiting the ED because of headache and 2) patients with a definite diagnosis of “tension-type headache” in the ED discharge report. We excluded patients with 1) some degree of uncertainty in the diagnosis, such as “possible” or “probable”; 2) another headache diagnosed at the same time; and 3) no available information in the patient chart.

We screened all the patients who visited the ED during the study period because of headache by using the ED database, which codifies patients by initial reason for consultation. We reviewed the digitalized reports and gathered the information from the discharge reports. We did not review any additional sources, and we did not evaluate any patients. The rationale for this was that we aimed to see if with the information present in the ED reports, TTH diagnosis was appropriate or not.

The included demographic variables were sex; age; and relevant prior medical history, including current or past cancer, pathology of the immune system, and prior headache history. Clinical variables included headache description, with special attention to the presence of any red flag, neurological examination and abnormal focal signs, vital signs and general examination.

### Study objectives

For the first objective, we reviewed the ICHD criteria for TTH^4–6^ (Table 1). We analysed whether each criterion was referenced in the report and if it was fulfilled or not. Criterion A, alluding to the number of episodes, was considered to be met for an infrequent tension-type headache diagnosis if the patient had a minimum of 10 episodes. We did not differentiate the ICHD edition in the manuscript, given that there were no differences between the TTH ICHD criteria in the second, third beta and third editions of the classification^4–6^ (Supplementary material).

**Table 1:** The International Classification of Headache Disorders criteria for Tension-type headache.

Criterion	Feature	Specific criterion
A.	Number of episodes	At least 10 episodes of headache.
B	Episode duration	Lasting from 30 minutes to seven days
C	Headache characteristics	At least two of the following four characteristics:
		1. Bilateral location
		2. Pressing or tightening (non-pulsating) quality
		3. Mild or moderate intensity
		4. Not aggravated by routine physical activity, such as walking or climbing stairs
D	Associated symptoms	Both of the following:
		1. no nausea or vomiting
		2. no more than one of photophobia or phonophobia
E	Exclusion	Not better accounted for by another ICHD-3 diagnosis

For the second objective, we analysed discrepancies concerning both anamnesis and prior medical history. Anamnesis discrepancies were classified into five groups:

1. Presence of *symptoms highly suggestive of another headache disorder*, including pulsating quality, presence of both photophobia and phonophobia, worsening with exercise, cervical topography, neuralgiform pain, or highly localized pain.
2. Presence of *abnormal neurological symptoms or signs*, such as aphasia, dysarthria, sensory disturbances, paresis, visual symptoms, vertigo, instability, and papilledema.
3. Presence of *red flags* related to the headache description, such as thunderclap onset, severe intensity (>9/10), progressive worsening, recent onset, worsening with Valsalva manoeuvre, precipitation by exercise, and refractoriness to appropriate treatment.
4. *Systemic symptoms*, such as fever, chest pain, abdominal pain, arthralgia, diarrhoea, urethral syndrome, and localized ocular pain.
5. *Close temporal relation* with an event able to produce headache, including cranial trauma, high blood pressure, lumbar puncture, and cranial surgery.

Discrepancies in *prior medical history* included prior headache disorders, cancer affecting encephalic structures, sinus disease, ophthalmological diseases able to produce ocular pain, sleep apnoea syndrome, and any other intra- or extracranial conditions able to produce headache.

For the third objective, two headache specialists (NGG, DGA) independently reviewed each case and analysed the information present in the discharge reports. With that information, when possible, patients were re-assessed according to ICHD-3 (77). In case of discrepancies, a third headache specialist (JPE) solved the disputes.

We also evaluated the management of patients. We determined the total duration of the emergency department stay, from when the patient was admitted to discharge. We analysed whether patients had been examined, specifically reviewing whether fundoscopy had been done. Complementary exam referrals were also addressed, including lumbar puncture, cranial tomography (CT), X-ray, and laboratory exams. Management at discharge was reviewed, inspecting if any treatment had been prescribed and if patients were referred to the neurological department. We compared whether patients who had undergone neurological exams or complementary exams had a longer stay and if they were more often diagnosed correctly.

The local ethics committee board approved the study. The study was performed according to the principles of the Declaration of Helsinki. Informed consent was obtained from all the participants.

### Statistics

Qualitative variables are presented as frequencies and percentages. Quantitative variables are presented as the means and standard deviations (sds) or medians and interquartile ranges (IQRs) in the case of a nonnormal distribution. Normality was tested by using the Kolmogorov-Smirnov test. The first three endpoints did not include statistical analysis and were based on descriptive qualitative data. As we analysed only patients with a definite TTH diagnosis, we only determined the positive predictive value of TTH diagnosis. In the analysis of the management of patients, for the comparison of qualitative variables, we used the chi squared test. When comparing two continuous variables, we used Student’s t test if a normal distribution was shown in the Kolmogorov-Smirnov test, and we used the Kruskal-Wallis test if the data were not normally distributed. We considered a p-value as significant if it was lower than 0.05 and specified degrees of freedom (df). In case of missing data, we performed complete-case analysis. We did not anticipate any sample size a priori but included all possible patients during the study period.

### Ethics approval and consent to participate

The Clinical Research Ethics Committee of Hospital Clínico San Carlos approved the study. Informed consent forms were obtained from the participants.

### Consent for publication

All authors gave their consent for publication.

## Results

During the study period, 2132 patients visited the ED because of headache. The inclusion/exclusion criteria were satisfied by 211 patients (9.9% of the total sample), and these patients were included in the study. The median age of the patients was 42.6 years [30.9–57.3], and 75.6% were female.

### Tension-type headache criteria

**Figure 1:**
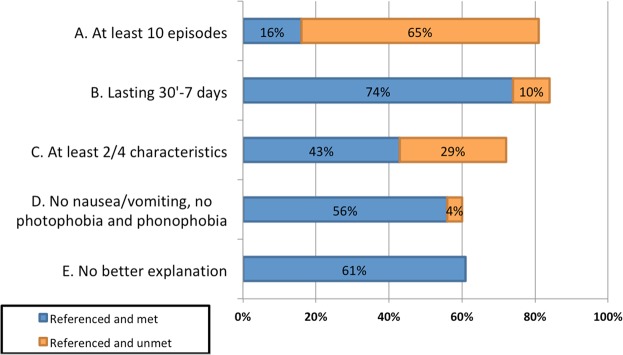
Percentage of patients in which every criterion is referenced; in blue referenced and met; in orange, referenced but unmet.

Only five patients fulfilled all ICHD criteria for TTH (2.4% of the included patients). The frequencies at which each criterion was referenced were as follows: criterion A was referenced in 81% of patients and fulfilled in 16% of patients, criterion B was referenced in 84% of patients and fulfilled in 74% of patients, criterion C was mentioned in 72% of patients and fulfilled in 43% of patients, and criterion D was referenced in 60% of patients and fulfilled in 56% of patients. Figure 1 shows the percentage of patients who fulfilled each criterion.

### Discrepancies in TTH diagnosis

There was at least one discrepancy in regard to anamnesis in 184 patients (87.2%). Symptoms suggestive of another headache disorder were present in 131 subjects (62.1%); 87 patients (41.2%) described other neurological symptoms; red flags were identified in 55 patients (26.1%); 57 subjects (27.0%) also reported systemic symptoms; and in 31 patients (14.6%), an event able to produce headache was described.

Concerning events related to headache onset, in 12 patients, traumatic injury to the head had occurred in the prior 7 days and was mentioned by patients; in 11 patients, an acute increase in blood pressure (BP) over 180/140 was documented, with cessation of headache after proper BP management; in three patients, headache started after dental manipulation with no prior history of headache; in two patients, headache began after the use of corticosteroids or amisulpride and olanzapine; in two patients, headache started after lumbar puncture; and in one patient, an intracranial aneurism was embolized the same day the headache started.

Regarding prior medical history, 44 patients (20.85%) had some condition able to produce headache. In 17 patients, a prior history of migraine was present. Those patients referred to an unchanged headache phenotype that was resistant to treatment; nine patients had acute sinusitis, four patients had painful ophthalmological conditions (glaucoma in two, ophthalmic herpes in one and acute uveitis in one), three patients had cerebral and arteriovenous malformation, two patients had a recent history of subarachnoid haemorrhage, two patients had a history of intracranial cerebrospinal fluid (CSF) hypotension, one patient had cerebellar haemangioblastoma, one patient had Erdheim-Chester disease, one patient had a history of temporal arteritis, one patient had a history of occipital neuralgia, one patient had sleep apnoea, one patient had intracranial aneurism and one patient had polycythaemia vera with hyperviscosity syndrome.

### Re-classification according to ICHD-3

**Figure 2:**
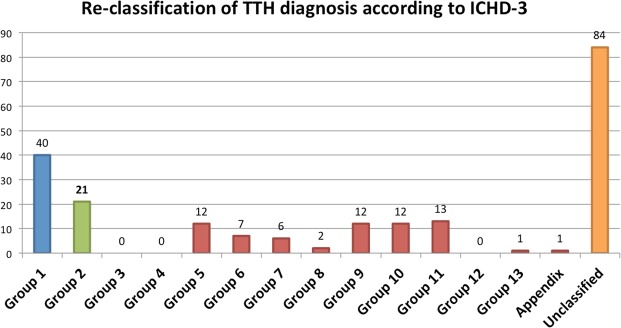
Number of patients re-classified to each ICHD-3 group. On the right column, patients unable to be re-classified.

After reviewing all the discharge reports, only 21 patients (9.9% of the included sample, 0.98% of the total sample) fulfilled the ICHD-3 criteria for tension-type headache (five) or probable tension-type headache (16). The positive predictive value of TTH diagnosis was 0.099. In 106 patients, another ICHD-3 diagnosis was met (50.2% of patients). In 64 patients, a secondary headache was the final diagnosis (30.3%), with 13 having high-risk secondary headaches (6.1%). Figure 2 and Table 2 show the number of patients encoded in each classification group and the specific diagnoses.

**Table 2:** Number and percentage of patients re-classified into each of the ICHD-3 diagnostic groups with specific diagnosis and codes.

Headache group	Number of cases (%)	Subtype
1. Migraine	40 (18.9%)	1.1 Migraine without aura (5)
		1.2 Migraine with aura (1)
		1.3 Chronic migraine (3)
		1.5 Probable migraine (31)
2. Tension-type headache	21 (10.0%)	2.2 Frequent tension-type headache (3)
		2.3 Chronic tension-type headache (2)
		2.4 Probable tension-type headache (16)
3. Trigeminal autonomic cephalalgias	0	0
4. Other primary headache disorders	0	0
5. Headache attributed to trauma injury to the head and/or neck	12 (5.7%)	Acute headache attributed to traumatic injury to the head (11)
		Acute headache attributed to whiplash (1)
6. Headache attributed to cranial and/or cervical vascular disorder	7 (3.3%)	6.2.4.2 Persistent headache attributed to past non-traumatic subarachnoid haemorrhage (2)
		6.3.2 Headache attributed to arteriovenous malformation (AVM) (3)
		6.7.1 Headache attributed to an intracranial endarterial procedure (1)
		6.4.1 Headache attributed to giant cell arteritis (1)
7. Headache attributed to non-vascular intracranial disorder	6 (2.8%)	7.2.1 Post-dural puncture headache (2)
		7.2.3 Headache attributed to spontaneous intracranial hypotension (2)
		7.4.1 Headache attributed to intracranial neoplasm (2)
8. Headache attributed to a substance or its withdrawal	2 (0.9%)	8.1.9 Headache attributed to occasional use of non-headache medication (2)
9. Headache attributed to infection	12 (5.7%)	9.2.2.1 Acute headache attributed to systemic viral infection (12)
10. Headache attributed to disorder of homeostasis	12 (5.7%)	10.3.2 Headache attributed to hypertensive crisis without hypertensive encephalopathy (11)
		10.1.4 Sleep apnoea headache (1)
11. Headache or facial pain attributed to disorder of the cranium, neck eyes, nose, sinuses, mouth or other facial or cervical structure	13 (6.2%)	11.3 Headache attributed to disorder of the eyes (2)
		11.3.3 Headache attributed to ocular inflammatory disorder (2)
		11.6 Headache attributed to disorder of the teeth
		11.5.1 Headache attributed to acute rhinosinusitis (3)
		11.5.2 Headache attributed to chronic or recurring rhinosinusitis (6)
12. Headache attributed to psychiatric disorder	0	0
13. Painful lesions of the cranial nerves and other facial pain	1 (0.5%)	Occipital neuralgia (1)
Appendix	1 (0.5%)	A10.8.2 Headache attributed to other metabolic or systemic disorder (polycythaemia vera, viscosity syndrome) (1)
Not classifiable	84 (39.8%)	

### Management of patients

The total duration of the ED visits, from admission to discharge, had a median length of 3.59 hours [IQR: 2.5–5.1], with a range between 0.35 and 19.10 hours. Neurological examination was described in the reports of 90.5% of patients and was abnormal in 6.8% of these patients. When the neurological exam was done, the duration of the ED visit was 4.0 hours vs. 3.9 hours when it was not done (Student’s t test, 22 df, p = 0.87). Fundoscopy was performed in 10.9% of patients, and the results were normal in all cases.

The diagnosis was more often appropriate in patients who underwent fundoscopy (chi-square test, 1 df, p = 0.004).

**Figure 3:**
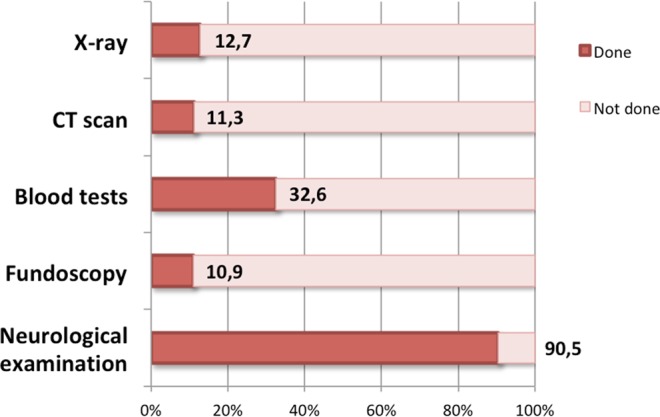
Percentage of patients that underwent each examination. CT scan (Cranial Tomography).

Regarding complementary exams, laboratory exams were performed in 32.6% of patients. Laboratory exams including erythrocyte sedimentation rate (ESR) or C-reactive protein (CRP) were performed in 48.6% of patients older than 65 years old. Cranial CT was performed in 11.3% of patients, and cervical or sinus X-ray was performed in 12.7% of patients. Only 2 CT exams were abnormal, one exhibiting a vascular malformation and the other with acute inflammation of the frontal sinuses. Figure 3 summarizes the percentage of patients who underwent each examination.

When complementary exams were performed, the mean duration of the stay increased from 3.28 to 4.94 hours (Student’s t test, 217 df, p < 0.001). Requests for complementary exams were not associated with a higher probability of proper diagnosis (chi-square, 1 df, p = 0.71). At discharge, 21.3% of patients were referred for a neurological examination. In 90% of the discharge reports, some acute medications were prescribed.

## Discussion

The present study was performed in the emergency department setting. We systematically analysed a series of patients with definite TTH diagnosis according to whether the diagnostic criteria were mentioned and fulfilled in the discharge report. We screened the reports for the presence of data that would contradict TTH diagnosis, and finally, we tried to reclassify patients based on the information provided in the reports.

The main findings of our study were that only a minority of patients, only 2.4% of the whole sample, fulfilled the ICHD criteria for TTH. In over 80% of patients, a discrepancy was found in the anamnesis, and in one-fifth of patients, prior medical history made TTH diagnosis unlikely. Finally, when we tried to reclassify patients, migraine diagnosis was two-times more frequent than TTH diagnosis. In almost 40% of patients, the information was not sufficient for a diagnosis, so in the best possible scenario, only half of patients with TTH diagnosis would have this condition, leading to a total percentage of 4.9% of the whole sample.

Concerning the ICHD criteria^6^, the most frequently mentioned criterion was duration, followed by number of episodes and phenotype. The most surprising data were that criterion A, describing the number of episodes, was mentioned in 81% of cases, but it was only fulfilled in 16% of cases. This fact may reflect the idea that many patients visit the emergency department due to recent-onset headaches. New onset headache and progressive worsening are two of the main red flags^18^. In patients with primary headaches, a previous history of similar unchanged attacks might constitute a green flag.

The ICHD Criteria for TTH, unchanged since the first edition^3^, attempt to differentiate TTH from the other main primary headache, migraine. The criteria include positive, negative and exclusion criteria. The positive criteria describe the typical characteristics of TTH: bilateral, pressing, and relatively mild. The negative criteria rule out the presence of typical migraine features, such as nausea, vomiting, photophobia and phonophobia or worsening as a result of activity. Finally, as in every ICHD diagnosis, the condition should not be better described by any other diagnosis. In the literature, some series have diagnosed TTH in 53% of patients with nausea^19^. Other series included patients who were diagnosed with TTH who had pulsating (23.8%) and hemicranial headaches (20.1%)^20^.

The typical TTH phenotype is probably the most unspecific, and many conditions might have similar features, such as migraine, hemicrania continua, primary cough headache, primary exercise headache, primary headache associated with sexual activity, external-pressure headache, hypnic headache, new daily persistent headache and the vast majority of secondary headache disorders^6,8^. Misdiagnosis might be related to the classification of patients based on pain phenotype; however, headache diagnosis should be performed by integrating prior medical history, headache anamnesis, presence of other symptoms and neurological examination, not solely by headache phenotype^8,21^.

The confusion surrounding TTH diagnosis might be partly influenced by the many names that have been used to describe this condition: tension headache, muscle contraction headache, psychomyogenic headache, stress headache, ordinary headache, essential headache, idiopathic headache, or psychogenic headache^3–6^. Some of these names may suggest a psychogenic cause. Currently, we consider stress and affective disorders a cause of worsening or a trigger rather than the cause of headache^22,23^.

Medical attention at the emergency department usually prioritizes the detection of life-threatening conditions. The frequent saturation, rush and wide variety of conditions complicate the thorough and meticulous anamnesis that headache disorders require. In our sample, 30% of patients had a secondary headache, and 5% of patients had a secondary headache with potential morbimortality. In a previous series, up to 10% of patients diagnosed with TTH had an abnormal examination^18^.

One of the biggest needs in the headache field is the development of reliable biomarkers. Unlike other painful syndromes, such as chest or abdominal pain, we still base our diagnosis on anamnesis and neurological examination^18^. Some rules^16^ and lists of red flags have been proposed to detect secondary headaches^24^. CT and lumbar puncture, although frequently requested^18,25,26^, do not always rule out many entities, such as cerebral venous sinus thrombosis, intracranial space-occupying lesions, or cerebrospinal fluid pressure disorders.

Headache is one of the leading reasons for consultation, accounting for 2.3% of all ED visits;^14,15,18^ therefore, all ED physicians should be trained in headache medicine. In our sample, just a minority of patients underwent fundoscopy, and in many cases, even plain X-ray was requested. The use of lab tests was not properly selected for elderly patients, as less than half of the patients had an ESR or CRP exam.

Sometimes an accurate diagnosis cannot be made; in our study, up to 38% of patients had an unspecific diagnosis, with this figure reaching 38–45% in the literature^24,27^. In those cases, it is important to note that declaring a primary headache disorder without certainty might implicate the end of the diagnostic work-up and a higher risk of complications. In case of doubt, final diagnosis should be “possible” or “headache not otherwise specified”^11,13,24^.

The consequences of inaccurate diagnosis are the risk of morbimortality among patients, inappropriate use of diagnostic resources, and the potential cost of establishing the proper diagnosis. Treatment of different secondary headaches differs widely, and in many conditions, prognosis is correlated with a prompt and proper treatment, as in the case of temporal arteritis, cerebral venous sinus thrombosis, or central nervous system infections^13^.

Although tension-type headache is supposed to be the most prevalent primary headache disorder^28,29^, it is seldom treated in headache outpatient clinics^30^, and some authors even suggest that patients diagnosed with TTH suffer from improperly diagnosed migraines in at least one-third of the cases^31,32^. Research on TTH and knowledge about its pathophysiology are also scarcer than those of other primary headaches, such as cluster headache or migraine^2,33^.

In many cases, TTH has been related to psychosocial factors^22^, and when patients complain of stress or mood disorders, TTH diagnosis is often made. It is well known that primary headache disorders are associated with significant personal, societal and familiar burdens^30^.

The pre-test probability of having TTH is 60–70%^1,2^ in the general population, but in the ED setting, it is important to clarify whether the headache that motivated the consult has changed, worsened progressively, resisted treatment or had new features. With regard to prior medical history, in our sample, one-fifth of the patients had conditions able to produce headaches, and 8% of the subjects were migraineurs. It is well known that chronic migraine exhibits a less typical phenotype, and some of the episodes might resemble TTH episodes^34^. In the diagnosis of chronic migraine, only 8 out of the minimum 15 headaches per month are needed to fulfil the migraine without or with aura criteria^6^.

The main limitations of the present study are the participation of a single centre, which might affect the generalizability of the results. Because of the design of the study, some data could have been asked and evaluated but not written; however, from a legal perspective, non-written data did not “occur”. The re-classification was performed without evaluating patients; therefore, there was potential for misclassification, and we used the current ICHD instead of the two editions valid during the study period (ICHD-2 and ICHD-3 beta). We did not follow up with patients to confirm the final diagnosis. The strengths of the study are the thorough review of every patient chart, the participation of headache experts and the three different analyses performed.

## Conclusion

In our sample, TTH was overdiagnosed in an emergency department, as only 2.4% of the patients fulfilled all ICHD criteria for TTH. Inconsistencies in prior medical history or anamnesis were present in the discharge reports in one-fifth and four-fifths of patients, respectively. Our analysis of medical records allowed us to reclassify these patients as having other primary or secondary headaches. Efforts to improve knowledge on headache disorders and ICHD are needed among ED physicians.

## Supplementary information

Supplementary material 1.

## Data Availability

Study material and supplementary material are available upon request from the corresponding author.
