# Supplementary material for: Tension-type headache in the Emergency Department Diagnosis and misdiagnosis: The TEDDi study
Source: Sci Rep. 2020 Feb 12;10:2446. doi: 10.1038/s41598-020-59171-4 (PMC7016102; doi:10.1038/s41598-020-59171-4)
Supplement: Supplementary file 1 — Supplementary material 1. [file 41598_2020_59171_MOESM1_ESM.docx]

**Tension-type headache in the Emergency Department Diagnosis and misdiagnosis: The TEDDi study**

David García-Azorín^1^, Mariam Fahrid-Zahram^2^, María Gutiérrez-Sánchez^1^, Nuria González-García^3^, Ángel L. Guerrero^1, 4, 5^, Jesus Porta-Etessam^3^.

**Supplementary material:**

**Supplementary material 1:** The international Classification of Headache Disorders criteria for Tension-type headache:

| Criterion | ICHD-1 (1988) | ICHD-2 (2004) | ICHD-3 beta (2013) | ICHD-3 (2018) |
| --- | --- | --- | --- | --- |
| A | Frequency criterion | Frequency criterion | Frequency criterion | Frequency criterion |
| B | Headache lasting from 30 minutes to 7 days | Headache lasting from 30 minutes to 7 days | Lasting from 30 minutes to 7 days. | Lasting from 30 minutes to 7 days. |
| C | Headache has at least two of the following characteristics:   1. Pressing / tightening (non-pulsating) quality 2. Mild or moderate intensity (may inhibit, but does not prohibit activities) 3. Bilateral location. 4. No aggravation by walking stairs or similar routine physical activity | Headache has at least two of the following characteristics:   1. Bilateral location. 2. Pressing / tightening (non-pulsating) quality 3. Mild or moderate intensity 4. Not aggravated by routine physical activity as walking or climbing stairs | At least two of the following four characteristics:   1. Bilateral location 2. Pressing or tightening (non-pulsating) quality 3. Mild or moderate intensity 4. Not aggravated by routine physical activity such as walking or climbing stairs | At least two of the following four characteristics:   1. Bilateral location 2. Pressing or tightening (non-pulsating) quality 3. Mild or moderate intensity 4. Not aggravated by routine physical activity such as walking or climbing stairs |
| D | Both of the following:   1. No nausea or vomiting (anorexia may occur) 2. Photophobia and phonophobia are absent, or one but not the other is present | Both of the following:   1. No nausea or vomiting (anorexia may occur) 2. No more than one of photophobia or phonophobia | Both of the following:   1. No nausea or vomiting 2. No more than one of photophobia or phonophobia | Both of the following:   1. No nausea or vomiting 2. No more than one of photophobia or phonophobia |
| E | At least one of the following:   1. History, physical and neurological examinations do not suggest secondary disorders. 2. History and/or physical and/or neurological examination do suggest such disorder, but is ruled out by appropriate investigations. 3. Such disorder is present, but tension-type headache does not occur for the first time in close temporal relation to the disorder. | Not attributed to another disorder | Not better accounted for by another ICHD-3 diagnosis | Not better accounted for by another ICHD-3 diagnosis |

References:

1. Headache Classification Committee of the International Headache Society. Classification and diagnostic criteria for headache disorders, cranial neuralgias and facial pain. Cephalalgia. 1988;8:suppl7:1-96.
2. Headache Classification Subcommittee of the International Headache Society. The International Classification of Headache Disorders, 2^nd^ Edition. Cephalalgia. 2004;24,supl 1:9-160.
3. Headache Classification Committee of the International Headache Society (IHS). The International Classification of Headache Disroders, 3^rd^ edition (beta version). Cephalalgia. 2013;33(9):629-808.
4. Headache Classification Committee of the International Headache Society (IHS). The International Classification of Headache Disorders, 3rd edition. Cephalalgia. 2018; 38:1–211.
